# Expression of *Barhl2* and its relationship with Pax6 expression in the forebrain of the mouse embryo

**DOI:** 10.1186/s12868-016-0311-6

**Published:** 2016-11-25

**Authors:** Elisa V. Parish, John O. Mason, David J. Price

**Affiliations:** Centre for Integrative Physiology, The University of Edinburgh, Hugh Robson Building, Edinburgh, EH8 9XD UK

**Keywords:** Mouse, Development, Thalamus, Forebrain, Gene expression, *Zona limitans intrathalamica*, Pax6, Barhl2

## Abstract

**Background:**

The transcription factor Barhl2 is an antiproneural transcription factor with roles in neuronal differentiation. The functions of its homologue in *Drosophila* development are better understood than its functions in mammalian brain development. Existing evidence suggests that its expression in the embryonic forebrain of the mouse is regional and may complement that of another transcription factor that is important for forebrain development, Pax6. The aim of this study is to provide a more detailed description of the *Barhl2* expression pattern in the embryonic forebrain than is currently available, to relate its expression domains to those of Pax6 and to examine the effects of Pax6 loss on *Barhl2* expression.

**Results:**

We found that *Barhl2* is expressed in the developing diencephalon from the time of anterior neural tube closure. Its expression initially overlaps that of Pax6 in a central region of the alar diencephalon but over the following days their domains of expression become complementary in most forebrain regions. The exceptions are the thalamus and pretectum, where countergradients of Pax6 and *Barhl2* expression are established by embryonic day 12.5, before overall Pax6 levels in these regions decline greatly while *Barhl2* levels remain relatively high. We found that *Barhl2* expression becomes upregulated in specifically the thalamus and pretectum in *Pax6*-null mice.

**Conclusions:**

The region-specific expression pattern of *Barhl2* makes it likely to be an important player in the development of region-specific differences in embryonic mouse forebrain. Repression of its expression in the thalamus and pretectum by Pax6 may be crucial for allowing proneural factors to promote normal neuronal differentiation in this region.

## Background

The development of the central nervous system depends on the actions and interactions of transcription factors and morphogens linked together in complex gene regulatory networks. These networks serve to finely control processes such as tissue patterning and neuronal subtype specification [1, 2]. The *bar homeobox*-*like* (*Barhl*) family of transcription factors, *Barhl1* and *Barhl2*, are the mammalian homologues of the *Drosophila bar homeobox* (*BarH*) transcription factors *BarH1* and *BarH2* [3]. *Barhl2* is strongly expressed in the proliferative zones of specific regions in the mammalian forebrain [4]. Its interactions with the many other transcription factors expressed in these regions are likely to be critical for normal forebrain development.

The proteins encoded by *bar* genes and their homologues in other species are characterised by the presence of a homeodomain along with either one or two FIL domains—DNA-binding regions that are rich in the amino acids phenylalanine (F), isoleucine (I), and leucine (L) [5]. Transcription factors containing FIL domains can act as transcriptional repressors [6] via a mechanism involving their recruitment of the *Drosophila* co-repressor *Groucho* or its homologues in other species [7–13]. The *Drosophila BarH* genes are known to prevent ectopic neurogenesis in the fly retina by inhibiting the expression of *atonal* (*ato*) [14], a proneural transcription factor featuring a basic helix–loop–helix (bHLH) motif. There is evidence that the actions of the mammalian *Barhl* genes are mediated at least in part by their regulation of atonal-related bHLH transcription factors, such as those of the *Neurogenin* (*Ngn*) family [5, 15, 16].

*Barhl2* plays roles in neuronal subtype specification in the vertebrate nervous system. In the retina, *Barhl2* is required for amacrine cell (AC) subtype specification. Loss of *Barhl2* leads to the specification of increased numbers of cholinergic ACs at the expense of glycinergic and GABAergic ACs [17], while the premature expression of *Barhl2* in the zebrafish retina induces the differentiation of GABAergic ACs at the expense of non-GABAergic ACs and photoreceptors [18]. In the mouse spinal cord *Barhl2* serves to specify dl1 interneuron subtype, and the loss of *Barhl2* leads to an increase in the number of contralaterally-projecting interneurons, with a reduction in the number that project ipsilaterally [19].

Studies in *Xenopus* have shown that the *Xenopus BarH2* homologue, *Xbarhl2* [5], promotes the formation of the *zona limitans intrathalamica* (ZLI) [20], a forebrain organizer region that patterns the diencephalon via the secretion of morphogens including Sonic hedgehog (Shh) [21, 22]. Another transcription factor, paired-box 6 (Pax6), has an opposite effect on the ZLI, limiting its size [22–24]. Published data on *Barhl2* expression, which is limited, suggests that it might complement that of Pax6 throughout much of the embryonic mouse forebrain with the possible exception of the thalamic ventricular zone, in which both genes appear to be strongly expressed at some embryonic stages [4, 25–27]. We carried out a comprehensive analysis of the forebrain expression of *Barhl2* in embryonic mice at a range of developmental stages, using qualitative and quantitative methods to examine its relationship with the expression of Pax6. We examined expression of *Barhl2* in the *Pax6*-null mutant mouse to test for a functional relationship between the expression patterns of *Barhl2* and Pax6.

## Methods

### Experimental animals and ethics statement

All experimental work was carried out in accordance with the UK Animals (Scientific Procedures) Act 1986 and UK Home Office guidelines [28]. All protocols were reviewed and approved by the named veterinary surgeons of the College of Medicine and Veterinary Medicine, the University of Edinburgh, prior to the commencement of experimental work.

Wild-type mice used were of the *Mus musculus* strain CD-1^®^ [29]. Timed matings were set up between CD-1^®^ males and females. The day on which a vaginal plug could be observed was taken to be embryonic day 0.5 (E0.5). Embryos were harvested at E8.5–E13.5. *Pax6*-null mice used were of the *Mus musculus* strain *Sey* ^*Ed*^ [30]. Crosses were set up between *Pax6* ^+/*Sey*^ males and *Pax6* ^+/*Sey*^ females to generate litters comprising *Pax6* ^+/+^, *Pax6* ^+/*Sey*^, and *Pax6* ^*Sey*/*Sey*^ embryos. Embryos were harvested at E11.5–E12.5. *Pax6* ^*Sey*/*Sey*^ embryos were identified by their lack of eyes.

### In situ hybridization

Harvested embryos were fixed in a solution of 4% paraformaldehyde (Fisher Scientific) in phosphate buffered saline (PBS) (Oxoid) at 4 °C overnight before being sucrose-sunk as previously described [31] and fixed in a 1:1 mixture of 30% sucrose solution in PBS optimal cutting temperature (OCT) medium (Sakura). For chromogenic in situ hybridisation embryos were then cryosectioned at a thickness of 10 µm before the protocol was performed as previously described [31]. For fluorescence in situ hybridization embryos were sectioned at a thickness of 16 µm before the protocol was performed as previously described [32].

The RNA riboprobe for *Pax6* was that described in Pinson et al. [33]. The RNA riboprobe for *Barhl2* [4] was a kind gift from Asuka Suzuki-Hirano and Tomomi Shimogori. The *Ngn2* probe was that described in Gradwohl et al. [34]. The *Shh* probe was that described in Echelard et al. [35].

### Immunohistochemistry

Following fluorescence in situ hybridization for *Barhl2*, antigen retrieval was performed by microwaving sections in a 10 mM aqueous solution of sodium citrate. Immunohistochemistry for Pax6 protein was performed as previously described [36]. The primary antibody used was rabbit Poly Pax6 (Covance Research Products) at a concentration of 1:400 in blocking solution. The secondary antibody used was goat anti-rabbit conjugated to Alexa-Fluor 488^®^ (Abcam) at a concentration of 1:400 in blocking solution. Anti-Nestin primary antibody was used at 1:40 (Becton–Dickinson).

### Imaging

Brightfield images were recorded with the Leica DMLB microscope and Leica Application Suite software. Fluorescence images were recorded using the Nikon A1R-FLIM confocal microscope and Nikon Elements software. The “grab large image free shape” function of elements was used to compile a tiled image from several square images recorded at different regions of the tissue section. For each of the three channels (Pax6 immunostaining at 488 nm, *Barhl2* in situ hybridization staining with cyanine-3-tyramide at 554 nm, DAPI staining of chromatin at 350 nm) a 12-bit greyscale image was recorded at a resolution of 1028 × 1028 pixels. Each set of three greyscale images was saved as a stack. All confocal images were recorded at the Image Analysis MultiPhoton and Confocal Technologies (IMPACT) imaging facility, The University of Edinburgh.

### Quantification of image data

The quantitative analysis was performed on images of coronal sections cut at the plane illustrated in Figs. 7 and 8A from each of three different embryos harvested at each of four developmental stages from E10.5 to E13.5 inclusive (12 embryos in total). The 12-bit greyscale images recorded for the Pax6 immunostaining channel (488 nm) and *Barhl2* in situ hybridisation channel (554 nm) were analysed using the Fiji software package [37]. For each image, the segmented line tool was used to draw a line through the Pax6 and *Barhl2*-expressing progenitor populations, parallel with the ventricular surface of the diencephalon from the dorsal midline to the ZLI (Fig. 8A). The intensity plot profile tool was then used to obtain average pixel greyscale values (ranging from 0 to 4096) along the line. The process was carried out on both left and right sides of the brain and the average values at each position along the line were plotted against distance from the dorsal midline. For images of embryonic tissue harvested at E10.5–E12.5, the line was 40 µm wide along its entire length. For embryos harvested at E13.5, the thickness of the neuroepithelium close to the dorsal midline had fallen below 40 µm, and so for this small region a 16 µm-wide line was used and intensity data from the two lines were subsequently combined. A linear regression trend line was calculated for each plot. The gradients of the trend lines for each of the three embryos analysed at each developmental stage were used to calculate the mean gradients of Pax6 and *Barhl2* expression for each stage.

## Results

### Expression of *Pax6* and *Barhl2* in the embryonic forebrain

**Fig. 1:**
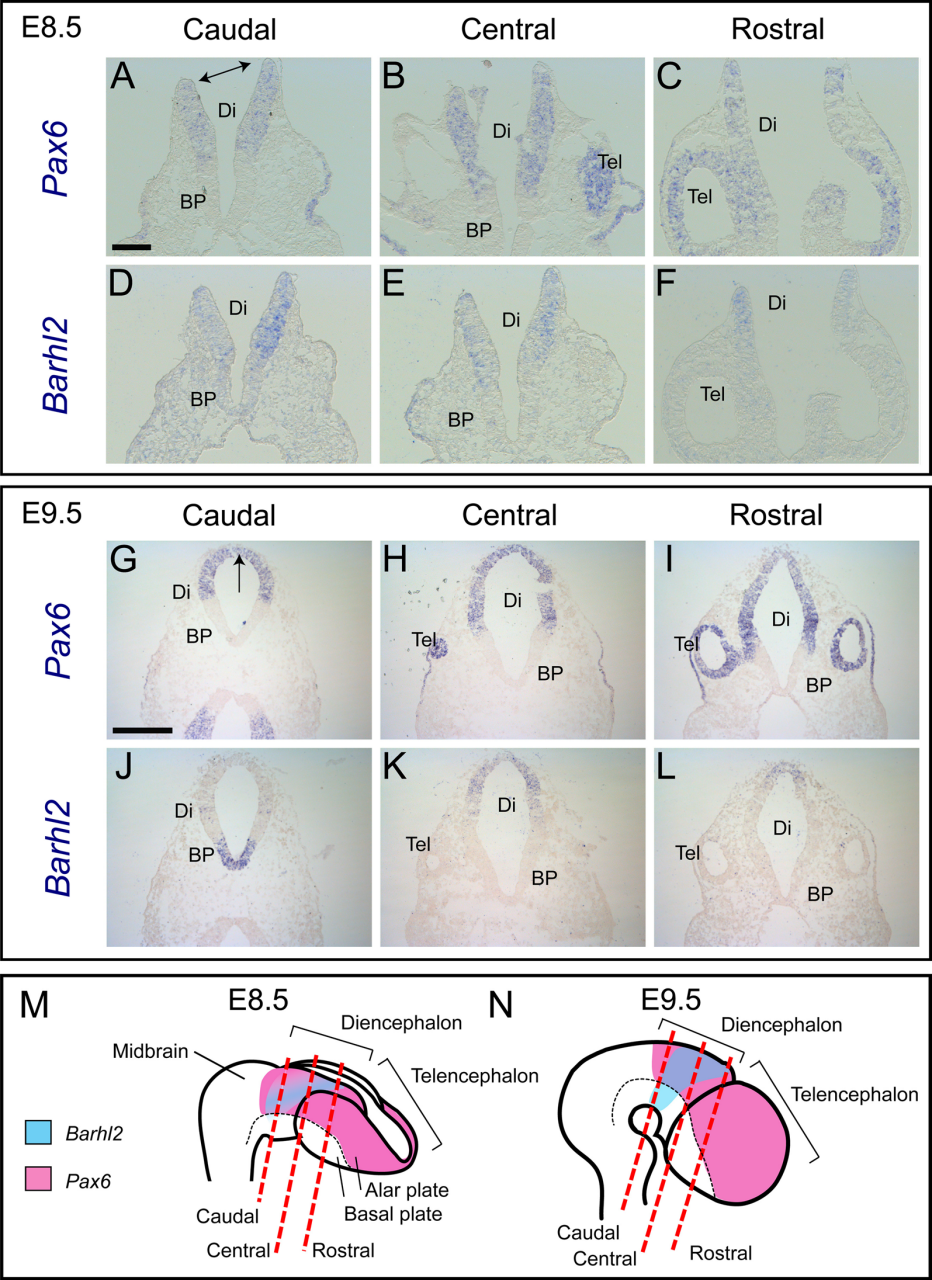
**A**–**L** In situ hybridization data for *Pax6* and *Barhl2* mRNA in adjacent sections cut from embryos at E8.5 and E9.5. **M**, **N** Schematics to illustrate the planes of the sections in **A**–**L** and to summarize the results. *Scale bar* for **A**–**F** 200 μm, **G**–**L** 500 μm. *Tel* telencephalon, *Di* diencephalon, *BP* basal plate

We first used chromogenic in situ hybridization to examine the expression of *Pax6* and *Barhl2* separately, on adjacent coronal sections through a series of embryonic brains of increasing age. At E8.5 (Fig. 1A–F), neural tube closure is not yet complete and the two dorsal edges of the neural tube can be observed prior to their fusing to form the roofplate (double-headed arrow, Fig. 1A). At this stage *Pax6* is expressed throughout most of the alar diencephalon and telencephalon (Fig. 1A–C) but is absent from their basal plate (Fig. 1A–C, summarized in Fig. 1M). *Barhl2* is also expressed throughout much of the diencephalon, with strongest expression in alar regions overlapping the middle of the diencephalic domain of *Pax6* expression (Fig. 1D–F, summarized in Fig. 1M). *Barhl2* expression is absent from the telencephalon (Fig. 1F).

At E9.5, following the closure of the neural tube and the formation of the roofplate (arrow, Fig. 1G), *Pax6* continues to be strongly expressed throughout alar regions of the forebrain but not the basal plate (Fig. 1G–I). *Barhl2* expression remains absent from the telencephalon (Fig. 1L, summarized in Fig. 1N). In the diencephalon, the *Barhl2* expression domain has consolidated into a band of neuroepithelium running from ventral (in the basal plate: Fig. 1J) to dorsal (Fig. 1K, L), flanked by regions of diencephalic neuroepithelium expressing little or no *Barhl2* (summarised in Fig. 1N). The alar component of this band overlaps a central strip of the diencephalic domain of *Pax6* expression.

**Fig. 2:**
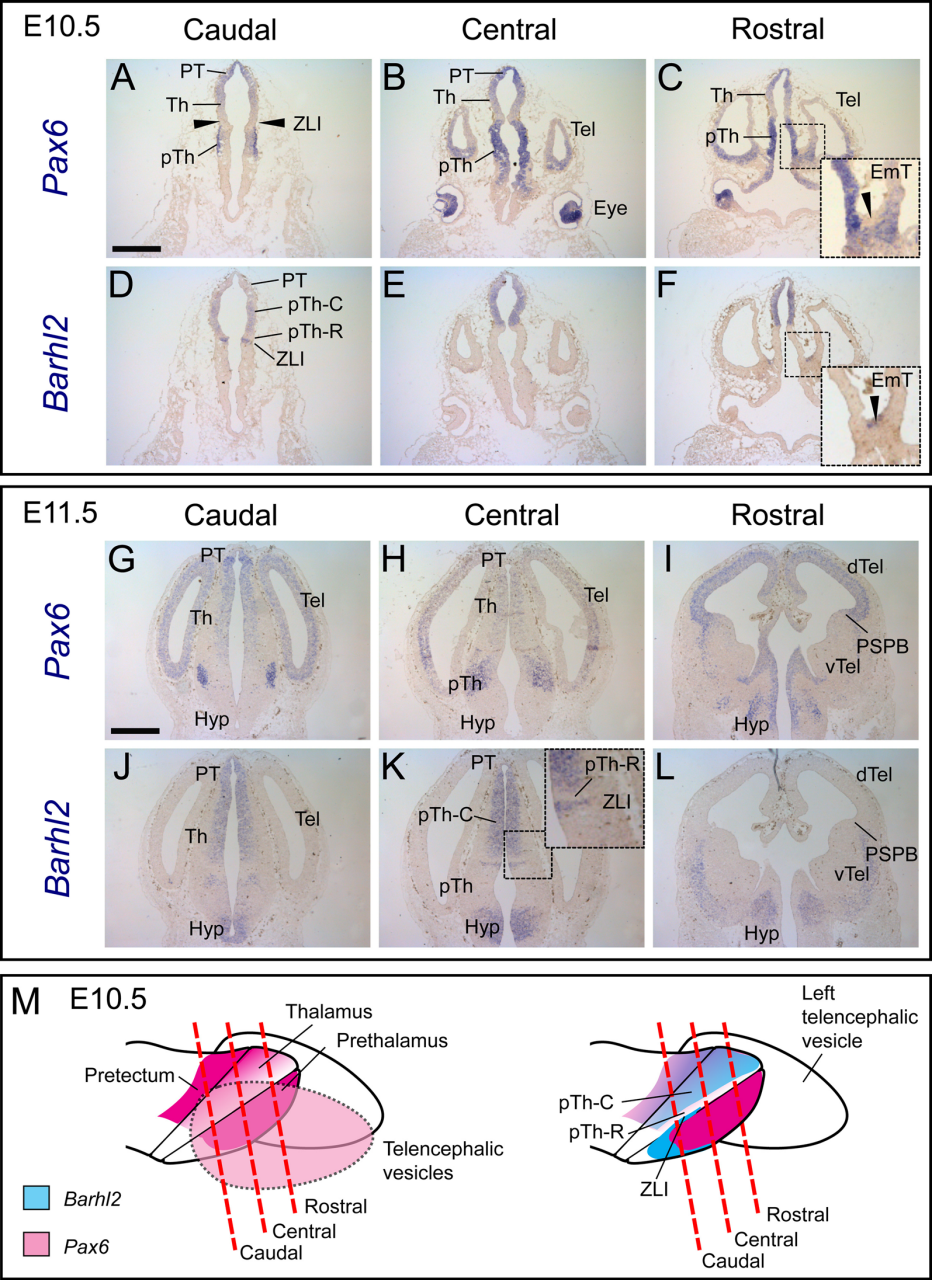
**A**–**L** In situ hybridization data for *Pax6* and *Barhl2* mRNA in adjacent sections cut from embryos at E10.5 and E11.5. **M** Schematics to illustrate the planes of the sections and to summarize results at E10.5. *Scale bars* 500 μm. *PT* pretectum, *Th* thalamus, *pTh* prethalamus, *ZLI zona limitans intrathalamica*, *EmT eminentia thalami*, *Hyp* hypothalamus, *PSPB* pallial–subpallial boundary, *Tel* telencephalon, *vTel* ventral telencephalon, *dTel* dorsal telencephalon

Previous work has shown that major regions of the diencephalon can be distinguished based on their morphology and their patterns of gene expression by E10.5. The ZLI is developing as a narrow Shh-expressing domain that spreads across the alar neural tube from ventral to dorsal, separating the prethalamus, which is rostral to the ZLI, from the thalamus, which is caudal to it [21, 38, 39]. *Pax6* expression levels now show greater regional variation within the alar forebrain (Fig. 2A–C). It is strongly expressed in the pretectum, prethalamus and telencephalon but is absent from the ZLI (arrowheads, Fig. 2A). It is also absent from the mantle cells of the developing *eminentia thalami* (arrowhead, Fig. 2C), which link the diencephalon and the telencephalon on each side of the brain. In the thalamus, *Pax6* expression levels are graded from low near the ZLI to high at the boundary with the pretectum (Fig. 2A–C). Strong *Pax6* expression can also be seen in the retina of the developing eye (Fig. 2B). These expression patterns are summarized in Fig. 2M.

Unlike *Pax6*, *Barhl2* is expressed within the developing ZLI itself at E10.5 (Fig. 2D). It is also expressed in the pretectum and throughout the majority of the thalamic ventricular zone, in the region of progenitor cells known as the pTh-C [22, 39, 40]. *Barhl2* is not expressed in a narrow strip of progenitor cells immediately caudal to the ZLI, a region known as the pTh-R [22, 39, 40] (Fig. 2D). *Barhl2* is also not expressed in the prethalamus (Fig. 2D–F), where *Pax6* expression is strong (Fig. 2A–C). In more rostral sections a domain of *Barhl2* expression can be seen within the mantle zone of the developing *eminentia thalami* (arrowhead, Fig. 2F), where *Pax6* is not expressed (Fig. 2C). *Barhl2* expression remains absent from the eye and the telencephalon (Fig. 2E, F). These expression patterns are summarized in Fig. 2M: essentially, significant complementarity between the patterns of expression of these two genes is emerging at E10.5, with residual overlap in the caudal diencephalon (pretectum and pTh-C).

By E11.5 (Fig. 2G–L), the complementarity of *Pax6* and *Barhl2* expression has become increasingly obvious throughout much of the forebrain. Whereas many telencephalic progenitor cells express *Pax6*, they do not express *Barhl2*. *Barhl2* is, however, now expressed by differentiating cells in the mantle zone of the ventral telencephalon, in regions where *Pax6* is not expressed (Fig. 2I, L). *Pax6* and *Barhl2* expression patterns in the hypothalamus also show striking complementarity (Fig. 2G–lL. In the diencephalon, *Pax6* continues to be strongly expressed within the prethalamus, where *Barhl2* is not expressed (Fig. 2H, K). The exception is the pretectum and the pTh-C domain of the thalamus, where both genes are expressed (Fig. 2G, H, J, K). *Barhl2* and *Pax6* both remain low or absent from the pTh-R (Fig. 2H, K) but only *Barhl2* is strongly expressed within the ZLI (Fig. 2K).

**Fig. 3:**
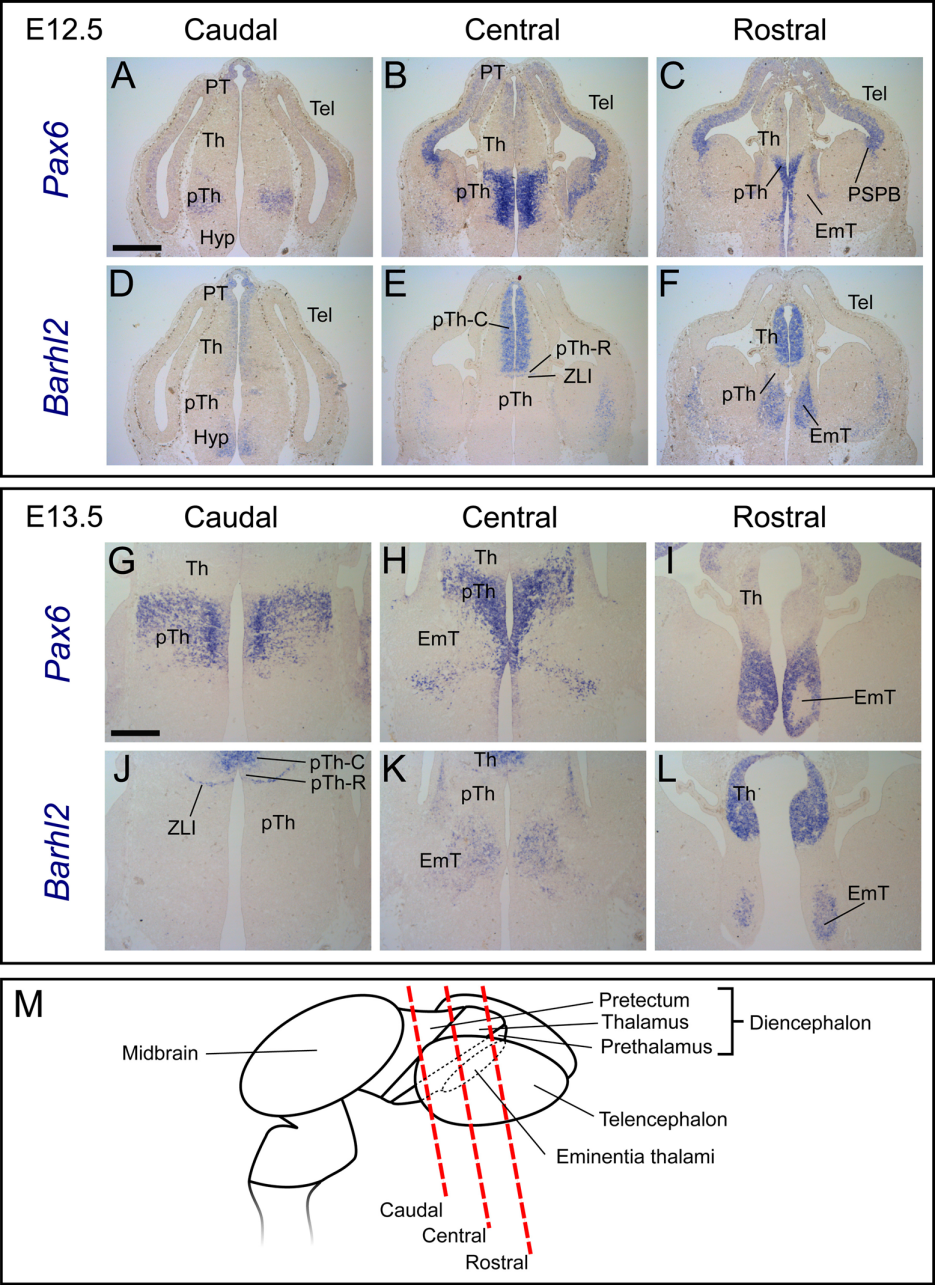
**A**–**L** In situ hybridization data for *Pax6* and *Barhl2* mRNA in adjacent sections cut from embryos at E12.5 and E13.5. **M** Schematic to illustrate the planes of the sections. *Scale bar* for **A**–**F** 500 μm, **G**–**L** 250 μm. *PT* pretectum, *Th* thalamus, *pTh* prethalamus, *Hyp* hypothalamus, *ZLI zona limitans intrathalamica*, *EmT eminentia thalami*, *PSPB* pallial–subpallial boundary, *Tel* telencephalon

The complementarity of *Pax6* and *Barhl2* expression patterns in the telencephalon, *eminentia thalami*, hypothalamus and prethalamus is well-developed at E12.5–E13.5 (Fig. 3). Neither gene is expressed specifically in progenitor or postmitotic zones: for example, *Pax6* is expressed in both zones in the prethalamus (Fig. 3A–C, G, H) but in progenitor zones alone within the dorsal telencephalon and thalamus (Fig. 3B). *Barhl2* is expressed in the progenitor zone of the thalamus (Fig. 3E) but in the postmitotic zone of the *eminentia thalami* (Fig. 3F, L). Overlap between *Pax6* and *Barhl2* expression patterns continues in the pretectum and pTh-C: *Pax6* is still expressed in a gradient but its levels are relatively low compared to those in other forebrain regions (Fig. 3B) whereas levels of *Barhl2* are as high or higher than those in other regions such as the ZLI (Fig. 3E, J) and *eminentia thalami* (Fig. 3F, L). *Barhl2* expression levels appear to be graded across pTh-C by E12.5, with levels increasing from caudal to rostral sections (Fig. 3D–F).

### Co-expression of Pax6 and *Barhl2* in the diencephalon

**Fig. 4:**
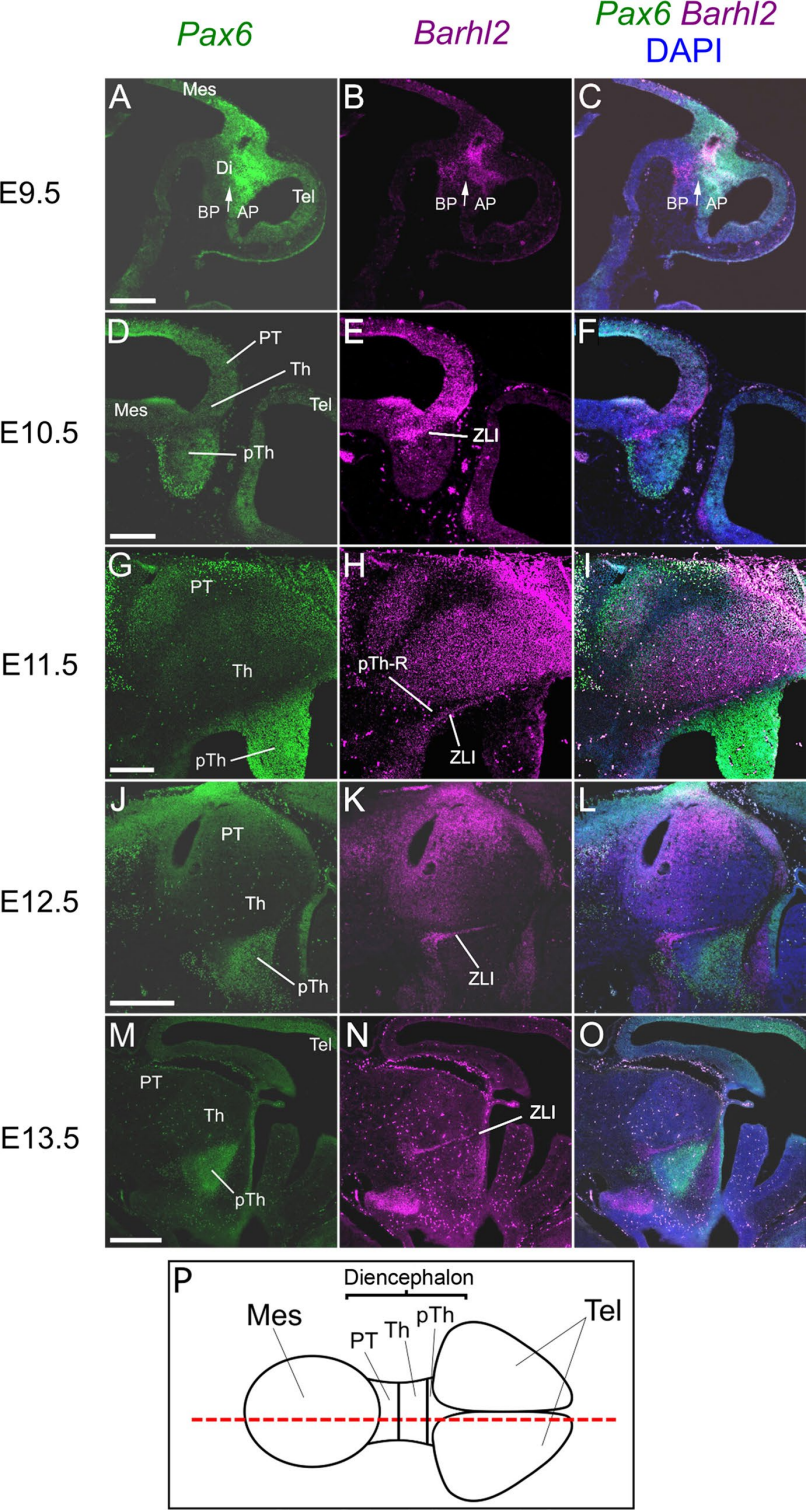
**A**–**O** Sagittal sections of embryos treated with immunostaining for Pax6 protein and in situ hybridization for *Barhl2* mRNA. **P** Schematic to illustrate the approximate plane of section. *Scale bars* for **A**–**I** 250 μm, **J**–**0** 500 μm. *Tel* telencephalon, *Di* diencephalon, *Mes* mesencephalon, *BP* basal plate, *AP* alar plate, *PT* pretectum, *Th* thalamus, *pTh* prethalamus, *ZLI zona limitans intrathalamica*

The analysis above indicates that diencephalic patterns of expression and co-expression of *Pax6* and *Barhl2* are complex and dynamic. To confirm and clarify the conclusions drawn from single-colour in situ hybridizations on adjacent coronal sections, we carried out fluorescence double-labelling with both probes on parasagittal sections of the brains of embryos of increasing age (Fig. 4).

**Fig. 5:**
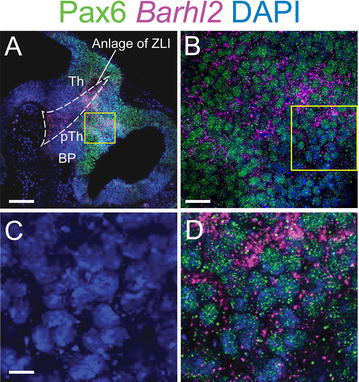
**A** Sagittal section of E9.5 embryo immunostained for Pax6 protein and in situ hybridization for *Barhl2* mRNA. *Scale bar* 200 μm. **B** Detail of area outlined in **A**. *Scale bar* 25 μm. **C**, **D** DAPI staining and triple-staining for DAPI, Pax6 and *Barhl2* within the prethalamus in the area outlined in **B**. *Scale bar* 10 μm

At E9.5, this analysis confirmed conclusions from coronal sections (summarised in Fig. 1N). *Pax6* is expressed throughout alar diencephalon and *Barhl2* is expressed in a narrower region, extending from the floorplate to the roofplate, whose alar domain overlaps a central portion of the diencephalic domain of *Pax6* expression (Fig. 4A–C). At high magnification expression of both Pax6 and Barhl2 could be seen in the presumptive ZLI (outlined area, Fig. 5A) and also in the region of prethalamic neuroepithelium directly rostral to it (Fig. 5B–D). *Barhl2* mRNA expression is detected primarily in the cytoplasm whereas Pax6 is located in the nucleus (Fig. 5B, D). In the alar prethalamic neuroepithelium, all cells expressed Pax6 at this age (Fig. 5C, D). Given the fact that *Barhl2* is present in surrounding cytoplasm between the Pax6-positive nuclei, we can deduce that many cells in this region co-express Pax6 and *Barhl2* (Fig. 5C, D).

At E10.5 the ZLI emerges as a thin *Barhl2*-positive, *Pax6*-negative domain between the *Barhl2*-positive thalamus and the prethalamus, which is now *Barhl2*-negative (Fig. 4D–F). This confirms conclusions summarized in Fig. 2M. The separation of the *Barhl2*-positive ZLI from the *Barhl2*-positive pTh-C by the pTh-R, a narrow strip of tissue expressing low or no *Pax6* and *Barhl2*, becomes clearer by E11.5 (Fig. 4G–I). The *Barhl2*-positive ZLI continues to be obvious at E12.5–13.5 (Fig. 4J–O).

**Fig. 6:**
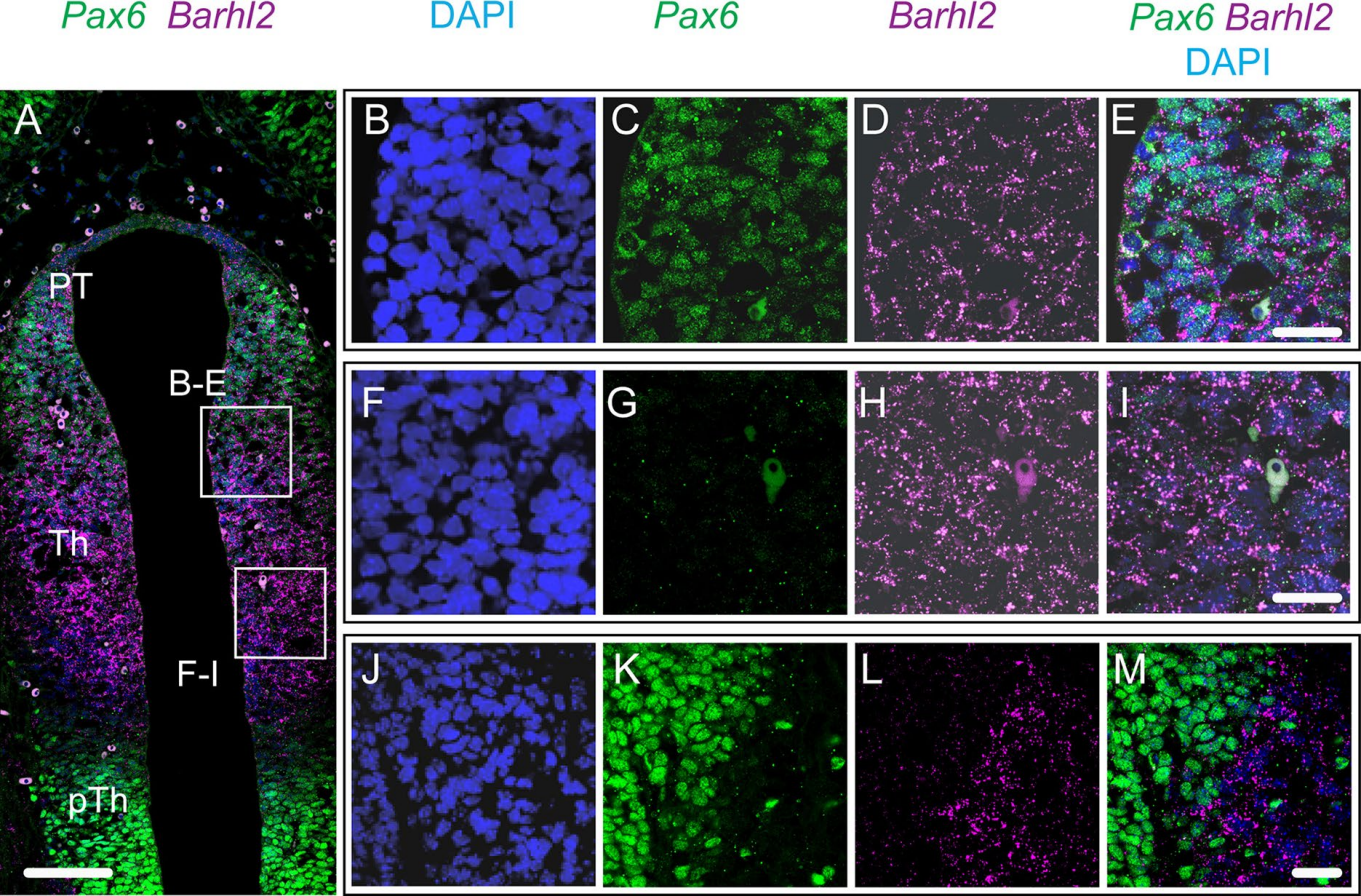
**A** Coronal section of E12.5 embryo treated with immunostaining for Pax6 protein and in situ hybridization for *Barhl2* mRNA. **B**–**E**, **F**–**I** Higher magnification of tissue outlined in **A**. **J**–**M** Detail of the *eminentia thalami*. *Scale bars* for **A** 100 μm, **B**–**M** 25 μm. *PT* pretectum, *Th* thalamus, *pTh* prethalamus

We next considered the relationship between the expression of *Pax6* and *Barhl2* in the pretectum and thalamus where, unusually, both are expressed in the same region for a prolonged period. We first considered whether Pax6 and *Barhl2* are expressed by the same cells in the pretectum and thalamus by double-labelling for Pax6 protein and *Barhl2* mRNA in the same coronal sections at E12.5 (Fig. 6). In these experiments we did not delineate the exact position of the boundary between the pretectum and thalamus, preferring to analyse the two together since the gradients of Pax6 and *Barhl2* expression were continuous across the two regions (Fig. 6A). As shown in Fig. 6A, C, G, cells express higher levels of Pax6 the closer they are to the pretectum. Close to the pretectum, almost all cells express detectable levels of Pax6 (Fig. 6B, C). These cells also stain for *Barhl2* in the cytoplasm around their Pax6-positive nuclei (Fig. 6D, E). This contrasts with other forebrain regions, such as the *eminentia thalami*, where Pax6-expressing and *Barhl2*-expressing cells are clearly segregated (Fig. 6J–M).

**Fig. 7:**
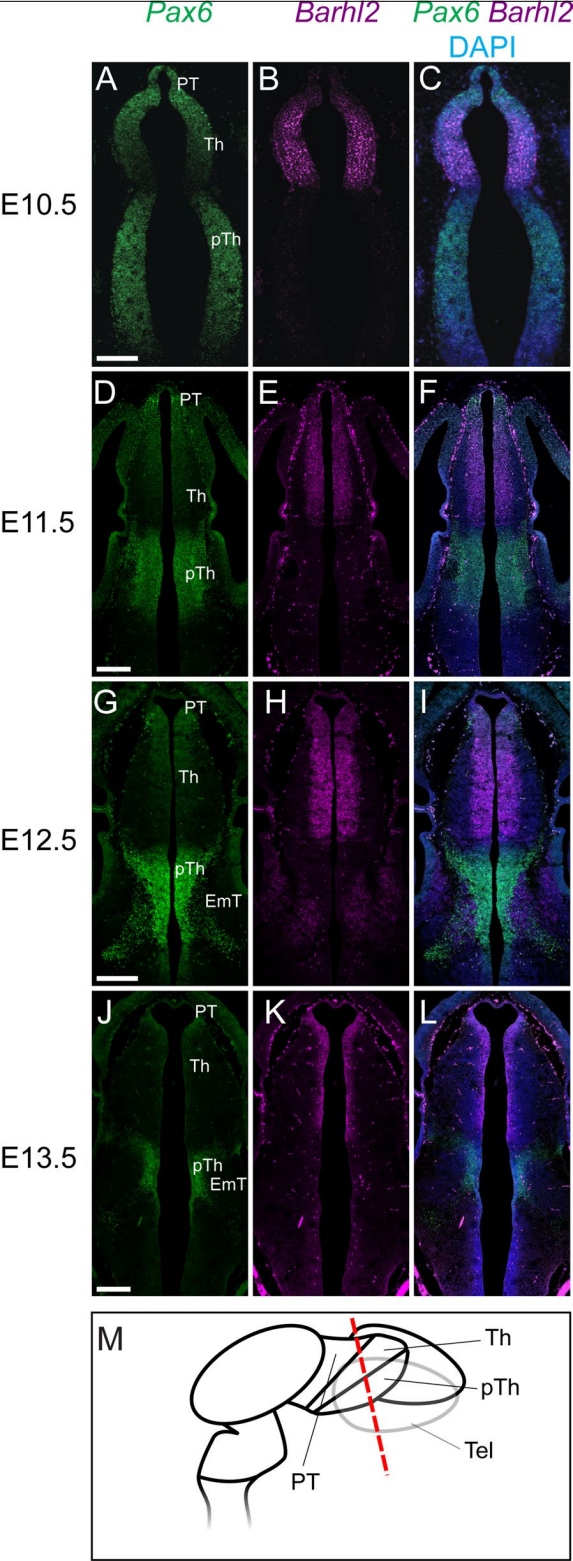
**A**–**L** Coronal sections of embryos treated with immunostaining for Pax6 protein and in situ hybridization for *Barhl2* mRNA. **M** Schematic to illustrate the approximate plane of section. *Scale bars* 500 μm. *PT* pretectum, *Th* thalamus, *pTh* prethalamus, *ZLI zona limitans intrathalamica*, *EmT eminentia thalami*, *Tel* telencephalon

To study the relationship between the gradient of Pax6 and expression levels of *Barhl2* across the thalamus, we examined coronal sections double-labelled with immunohistochemistry for Pax6 and fluorescence in situ hybridization for *Barhl2* (Fig. 7). The plane at which these sections were cut (Fig. 7M) offers a clear view of the gradient of Pax6 expression (Fig. 7A, D, G, J) and, therefore, the opportunity to correlate this gradient with variations in *Barhl2* expression. In the examples shown in Fig. 7 (and also that shown in Fig. 6) there is evidence of countergradients of *Barhl2*, with levels increasing towards the ZLI, at E10.5–E12.5. By E13.5, Pax6 levels in the thalamus are very low and there is no longer any obvious gradient of *Barhl2*, which is relatively strongly expressed in the ventricular zone of the thalamus (Fig. 7J–L).

**Fig. 8:**
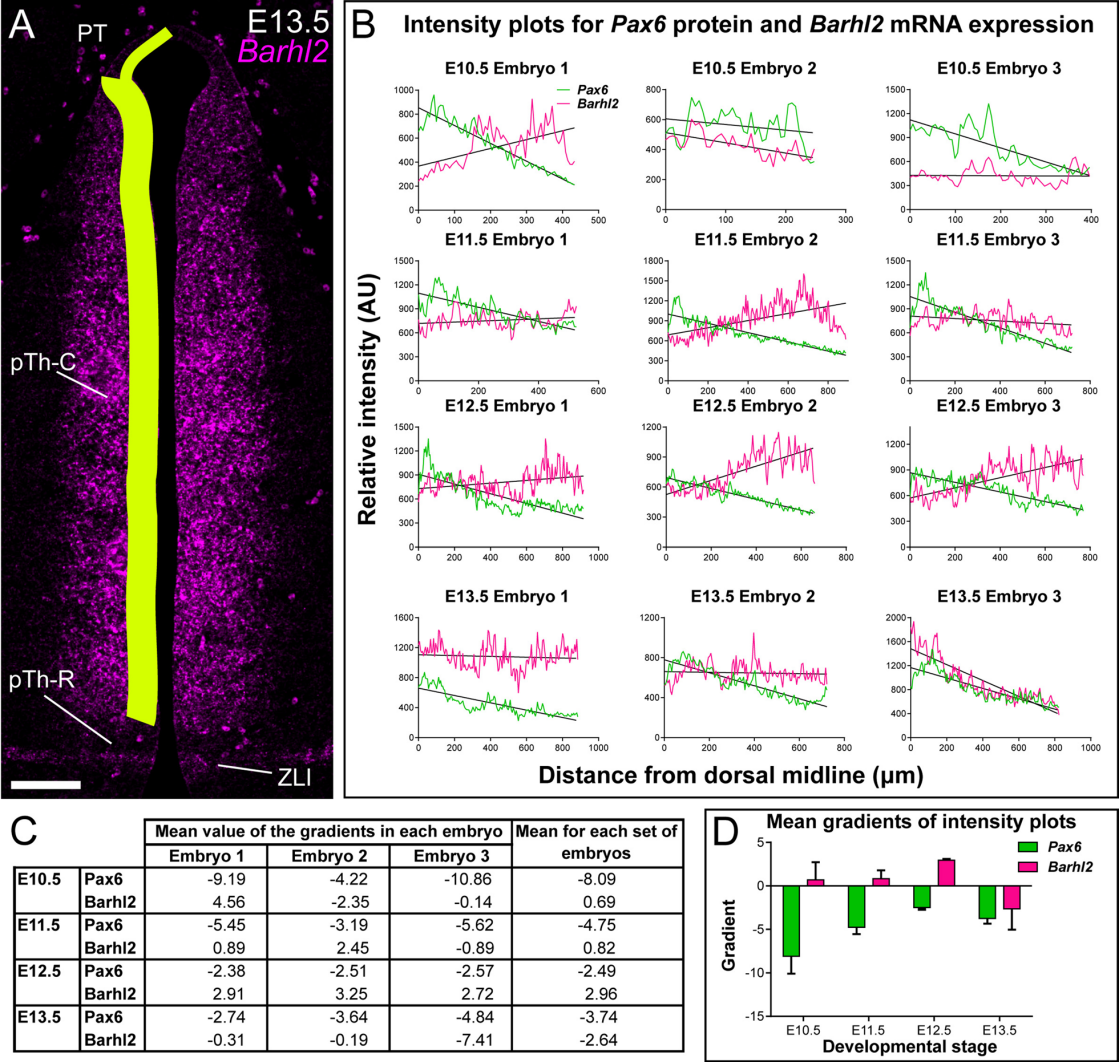
**A** Schematic to illustrate the area of the ventricular zone across which the intensity plots were calculated. *Scale bar* 100 μm. **B** Intensity plots for 12 embryos, three at each of four ages from E10.5 to E13.5, with linear regression trend lines. **C** Table of the gradients of the trend lines for each developmental stage, and the mean gradient for each set of three embryos. **D** Bar chart of the mean values of the gradients recorded for each group of three embryos, with standard errors. *PT* pretectum, *ZLI zona limitans intrathalamica*, *dpc* days post conception

To gain more objective data on these countergradients and their variation between embryos of the same and different ages, we quantified the levels of Pax6 and *Barhl2* expression in three embryos at each of four ages, E10.5, E11.5, E12.5 and E13.5 (Fig. 8).

At all the ages studied—even at E13.5, when Pax6 levels are overall low throughout the thalamus (Fig. 7J)—the intensity of staining for Pax6 showed a consistently negative correlation with distance from pretectum to ZLI (Fig. 8B–D). This is shown by the green lines and the mean gradients for each individual embryo (Fig. 8B, C) and by relatively low variance around the means at each age (green bars in Fig. 8D). At E12.5, all three embryos show a countergradient of *Barhl2* (Fig. 8B–D), as shown by the magenta lines and the mean gradients for each E12.5 embryo in Fig. 8B, C and by the low variance around the mean at E12.5 (third magenta bar in Fig. 8D). At earlier ages, clear gradients of *Barhl2* expression were not always detected, although where strong gradients were detected they ran counter to those of Pax6 (Fig. 8B, C). At the latest age examined, E13.5, when Pax6 levels are generally very low (Fig. 7J), no countergradients were observed (Fig. 8B–D). These data suggest that an inverse relationship between thalamic gradients of Pax6 and *Barhl2* becomes established over the 2 days between E10.5 and E12.5, with variability in the timing of its emergence between individuals. The gradient of Pax6 appears to be established robustly before that of Barhl2.

### Expression of *Barhl2* in the Pax6-null forebrain

**Fig. 9:**
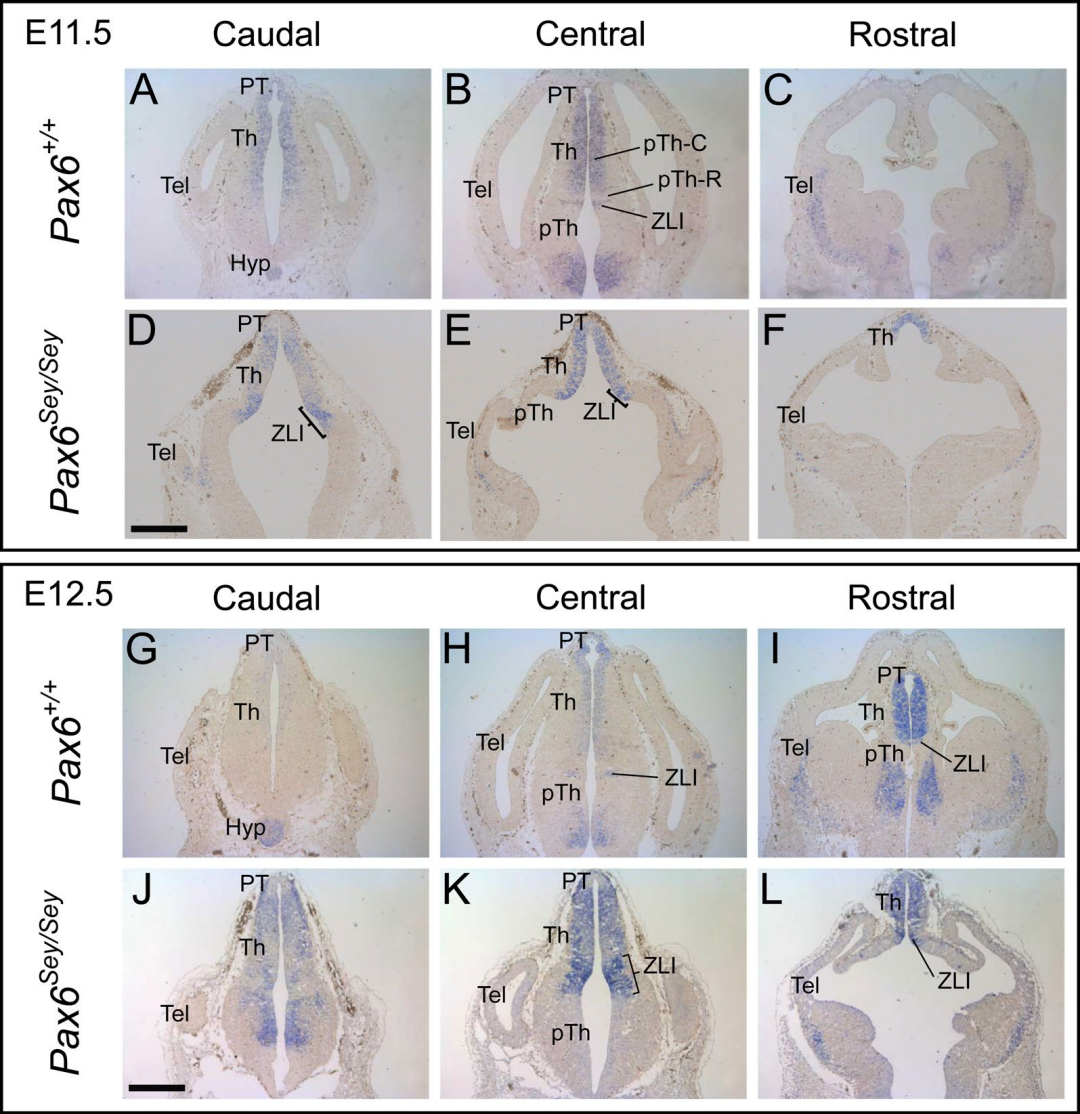
**A**–**F** In situ hybridization for *Barhl2* mRNA on comparable sections from *Pax6* ^+/+^ and *Pax6* ^*Sey*/*Sey*^ embryos at E11.5. **G**–**L** In situ hybridization for *Barhl2* mRNA on comparable sections from *Pax6* ^+/+^ and *Pax6* ^*Sey*/*Sey*^ embryos at E12.5. *PT* pretectum, *Th* thalamus, *pTh* prethalamus, *ZLI zona limitans intrathalamica*, *EmT eminentia thalami*, *Tel* telencephalon, *Hyp* hypothalamus. *Scale bars* 500 μm

The findings above suggested that Pax6 might repress the forebrain expression of *Barhl2*. In order to investigate this possibility further we performed in situ hybridization for *Barhl2* mRNA on cryosections from *Pax6* ^*Sey*/*Sey*^ forebrains (Fig. 9).

**Fig. 10:**
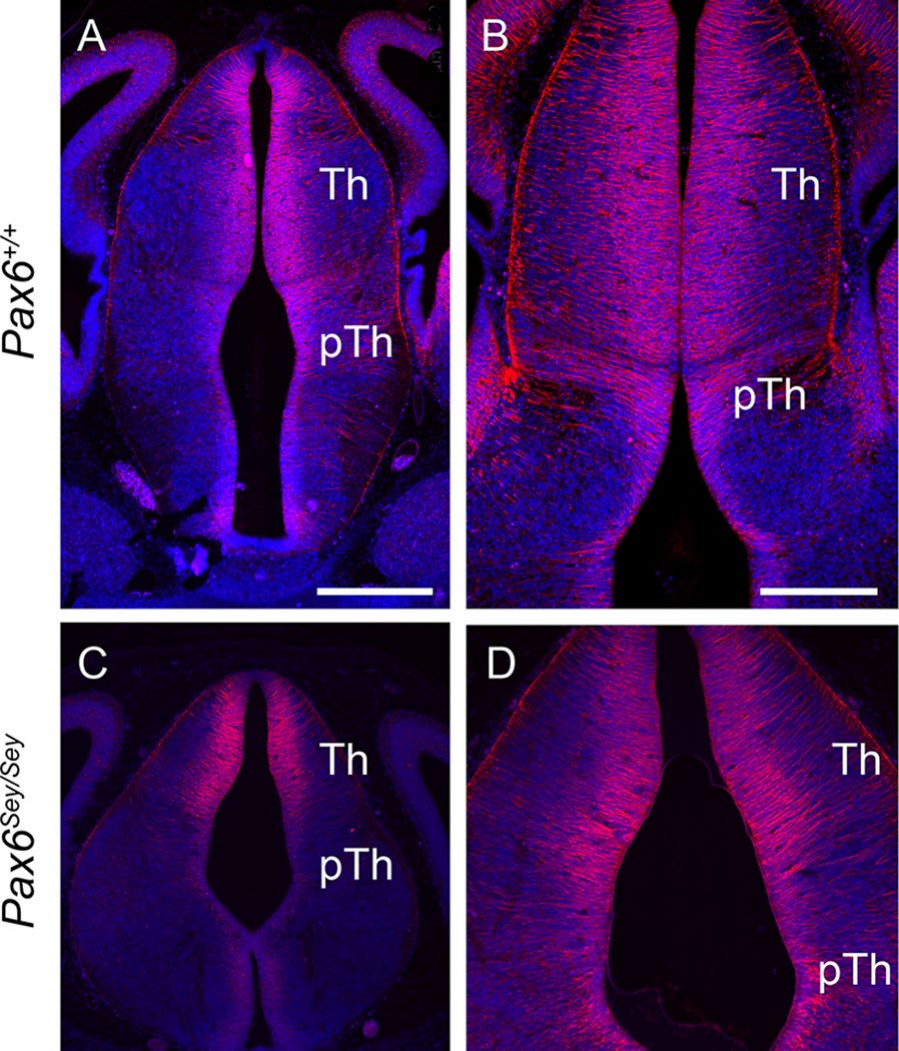
Immunohistochemistry for Nestin on sections of the E13.5 *Pax6* ^+/+^ and *Pax6* ^*Sey*/*Sey*^ diencephalon. *Th* thalamus, *pTH* prethalamus. *Scale bars* 500 µm in **A**, **C** and 250 µm in **B**, **D**

As has been described before, the morphology of the *Pax6* ^*Sey*/*Sey*^ mutant forebrain differs from that of the wild-type. The ZLI is expanded [22–24], much of the neuroepithelium is reduced in thickness [41, 42] and the third ventricle expands laterally [43] as a result of two diencephalic structures, the paraventricular nucleus and the caudal *zona incerta*, failing to develop correctly [44]. Despite these differences in morphology, structures such as the thalamus and prethalamus can be distinguished in both the wild type and mutant forebrain [22, 44], making it possible to compare the expression of *Barhl2* in these regions. Nestin staining for radial glial cells in the ventricular zone [45] was similar in *Pax6* ^+/+^ and *Pax6* ^*Sey*/*Sey*^ embryos and indicated that there was no major change in the depth of the ventricular zone in mutants (Fig. 10).

In the E11.5–E12.5 *Pax6* ^+/+^ diencephalon the domain of *Barhl2* within the pTh-C was restricted to the ventricular zone (Fig. 9A, B, G, H). In the *Pax6* ^*Sey*/*Sey*^ mutant the thalamic *Barhl2* domain spanned all or most of the mediolateral width of the thalamic neuroepithelium, most likely due to the absence of normal mantle zone development (Fig. 9D, E, J, K). *Barhl2* was expressed within the expanded ZLI of *Pax6* ^*Sey*/*Sey*^ mutants. The most striking difference between *Barhl2* expression in *Pax6* ^+/+^ and *Pax6* ^*Sey*/*Sey*^ diencephalon was its elevated expression in the thalamus and pretectum of mutants at E12.5, but not earlier. The pTh-R was clearer in the *Pax6* ^+/+^ diencephalon, where it was *Barhl2*-negative, than in the *Pax6* ^*Sey*/*Sey*^ diencephalon, where it showed weaker *Barhl2* expression than surrounding pTH-C and ZLI (Fig. 9B, E, H, K). The pattern of *Barhl2* expression in other forebrain regions appeared similar in both genotypes at E11.5–E12.5 (allowing for the morphological differences). The prethalamus, dorsal telencephalon and much of the ventral telencephalon (with the exception of ventro-laterally positioned cells which expressed *Barhl2* in both genotypes; Fig. 9C, F, I, L) remained negative for *Barhl2* in the mutants. We conclude that the loss of Pax6 causes increased *Barhl2* specifically in the pretectum and thalamus, where their co-expression is normally prolonged.

### Comparison of *Barhl2* and *Ngn2* expression in the embryonic forebrain

**Fig. 11:**
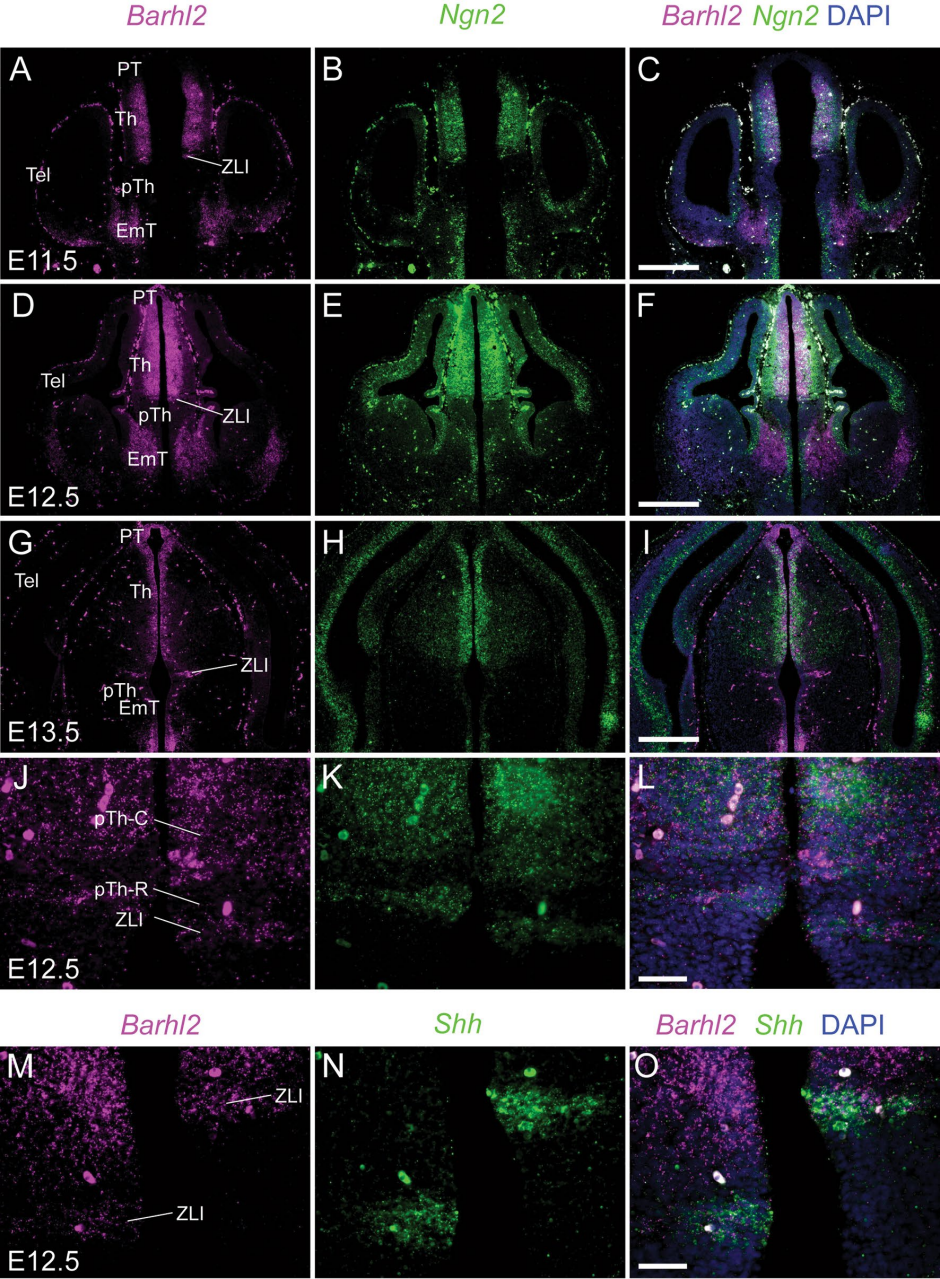
**A**–**L** In situ hybridization for *Barhl2* and *Ngn2* mRNA on coronal sections from *Pax6* ^+/+^ embryos at E11.5–E13.5. *Scale bars* for **A**–**I** 500 μm, **J**–**L** 50 μm. **M**–**O** In situ hybridization for *Barhl2* and *Shh* mRNA, high magnification image showing detail of the ZLI. *Scale bar* 50 μm. *PT* pretectum, *Th* thalamus, *pTh* prethalamus, *ZLI zona limitans intrathalamica*, *EmT eminentia thalami*, *Tel* telencephalon, *EmT eminentia thalami*

*Barhl2* has been suggested as an inhibitor of the expression of bHLH transcription factors. We compared its diencephalic expression to that of the bHLH transcription factor *Neurogenin2* (*Ngn2*) using double fluorescence in situ hybridization with riboprobes for *Barhl2* and *Ngn2* on sections cut at the level of the central diencephalon from embryos at E11.5, E12.5 and E13.5 (Fig. 11). In the telencephalon and the eminentia thalami, expression of *Barhl2* complemented that of *Ngn2* (Fig. 11C, F, I). *Ngn2* was expressed in the dorsal telencephalon and the proliferative zone of the eminentia thalami. *Barhl2*, on the other hand, was not expressed in dorsal telencephalon and was expressed in the differentiating but not the proliferating zone of the eminentia thalami.

In the diencephalon, however, the expression patterns of *Barhl2* and *Ngn2* were similar. Neither *Barhl2* nor *Ngn2* was expressed in the prethalamus and the pTh-R, while both were expressed in the ZLI and pTh-C (Fig. 11A–L). Within the ZLI, *Barhl2* expression corresponded with that of *Ngn2*, which is known to be expressed mainly in the more caudal region of the ZLI [22]. To confirm the location of *Barhl2* expression in the ZLI, double fluorescence in situ hybridization was performed using riboprobes for *Barhl2* and *Shh*, the definitive marker of the entire ZLI [21, 22]. The domain of *Barhl2* was slightly narrower than that of *Shh* within the ZLI and was centred caudally, as anticipated (Fig. 11M–O). In the thalamus and pretectum, *Barhl2* expression was confined to the proliferative zone whereas *Ngn2* expression extended through the proliferative zone and into the overlying differentiating mantle zone.

## Discussion

### Evidence for interactions between *Barhl2* and Pax6

The expression of *Barhl2* complements that of Pax6 in the telencephalon and eminentia thalami from early stages of its development, but the expression domains of *Barhl2* and Pax6 overlap in diencephalic regions. Early in development, there is co-expression of the two genes from prethalamus to pretectum, but *Barhl2* expression is rapidly turned off in prethalamus, where Pax6 levels remain high. Co-expression persists in the thalamus and pretectum, where Pax6 and *Barhl2* develop countergradients. Our data suggest that a robust gradient of Pax6 expression across this region predates the establishment of a robust countergradient of *Barhl2* expression. Soon after consistent countergradients are established, Pax6 levels become undetectable across most of this region, while *Barhl2* levels remain high. These results suggested that Pax6 expression might have a repressive effect on *Barhl2* expression. This might be direct or indirect. A screen designed to predict Pax6 target genes identified 13 potential Pax6 binding sites around the transcription start site of *Barhl2*, suggesting that Pax6 protein may be capable of exerting a direct effect on the expression of *Barhl2* mRNA [46].

We found that the absence of Pax6 did not induce *Barhl2* expression in telencephalic regions that normally express Pax6 but not *Barhl2*. Nor did the absence of Pax6 prevent the loss of *Barhl2* expression from the prethalamus. Absence of Pax6 did, however, increase *Barhl2* expression in the thalamus and pretectum. This interesting finding suggests a functional interaction between Pax6 and *Barhl2* only in the region of the forebrain in which co-expression of the two genes persists much longer than in any other region. Whereas factors other than Pax6 appear to control the overall expression patterns of *Barhl2*, Pax6 is likely to play an important part in modulating the expression levels of *Barhl2* in just this one specific part of the forebrain. Our findings indicated that absence of Pax6 led to *Barhl2* upregulation in thalamus and pretectum after E11.5, suggesting that Pax6 exerts its repressive effect on *Barhl2* only once the production of neurons is getting underway. The importance of this might be to limit the anti-proneural activity of *Barhl2*, reducing its ability to interfere with the proneural activity of factors such as Ngn2. It is possible that proneural inhibition by unfettered upregulation of *Barhl2* expression in the absence of Pax6 is a cause of the reduced size of the differentiating (mantle) zone in *Pax6*-null mutants.

Previous studies have provided limited evidence for the possibility that *Barhl2* and Pax6 might be mutually repressive. One study reported an expansion of the Pax6 expression domain in response to a morpholino knockdown of *Xbarhl2* in the Xenopus embryo [47]. Further work is needed to test whether loss of Barhl2 in mice affects the expression of Pax6.

The relationship between *Barhl2* and Pax6 expression in the pretectum and thalamus may differ from that observed in other forebrain regions for many reasons. The regions of neuroepithelium fated to become the thalamus and prethalamus are established prior to ZLI formation and differ significantly in molecular character from the forebrain regions rostral to the ZLI [48]. The thalamic anlage expresses both *Irx3* and *Pax6*, which together confer competence to develop into the thalamus in response to the *Shh* signal [37]. The prethalamic anlage strongly expresses *Pax6* but not *Irx3* [36] and also expresses *Six3*, which confers competence on the neuroepithelium to develop into more rostral forebrain structures [42]. Both regions of neuroepithelium are competent to respond to secreted signals from the ZLI [21], but respond in different ways, and signalling from the ZLI therefore exerts changes in gene expression in an asymmetric fashion [39]. These differences in molecular character may also influence the degree to which *Pax6* and *Barhl2* can be co-expressed.

### *Barhl2* and Pax6 in ZLI development

The ZLI is a thin Shh-expressing strip of tissue that separates the thalamus and prethalamus. Morpholino knockdown of *Barhl2* expression in *Xenopus* results in a failure of the ZLI to develop [20], while the loss of functional Pax6 in mice causes the ZLI to undergo an expansion along the rostrocaudal axis of the diencephalon [22–24]. Together these findings suggest that *Barhl2* is required for ZLI initiation, while *Pax6* may play a later role in the shaping of the ZLI. It is possible that Pax6’s inhibition of *Barhl2* might contribute to its role in limiting the region of diencephalon that is competent to develop into the ZLI. In the developing *Drosophila* retina Hh acts upstream of BarH1 and BarH2 and is required to initiate their expression [49]. In the vertebrate forebrain the need for *Barhl2* in the induction of the ZLI suggests that *Barhl2* may act upstream of Shh in this process and may be required to induce Shh expression. Alternatively, *Barhl2* may be required to render a central region of diencephalic neuroepithelium competent to develop into ZLI.

While *Barhl2* is expressed in the anlage of the ZLI, and later within the mature ZLI itself, it is not expressed throughout the whole of the mature ZLI. Its expression is confined to the more caudal, *Ngn2*-expressing region. It is not clear what function *Barhl2* serves in the mature ZLI, if any, and why it is not expressed throughout the entire ZLI.

## Conclusions

The expression of *Barhl2* in the thalamus and pretectum is related to, and regulated by, the expression of Pax6. *Pax6* is known to be required for normal diencephalic development. The findings presented here suggest that some of its actions might be mediated by its maintenance of a repressive influence over *Barhl2* expression in the thalamus and pretectum.

## Abbreviations

bHLH: basic helix–loop–helix
AC: amacrine cell
PBS: phosphate-buffered saline
OCT: optimal cutting temperature
Tel: telencephalon
Di: diencephalon
Mes: mesencephalon
Rh: rhombencephalon
SC: spinal cord
PT: pretectum
Th: thalamus
pTh: prethalamus
ZLI: *zona limitans intrathalamica*
EmT: *eminentia thalami*
Hyp: hypothalamus
vTel: ventral telencephalon
dTel: dorsal telencephalon
PSPB: pallial–subpallial boundary
BP: basal plate
AP: alar plate
dpc: days post conception
